# Deficiency in Origin Licensing Proteins Impairs Cilia Formation: Implications for the Aetiology of Meier-Gorlin Syndrome

**DOI:** 10.1371/journal.pgen.1003360

**Published:** 2013-03-14

**Authors:** Tom Stiff, Meryem Alagoz, Diana Alcantara, Emily Outwin, Han G. Brunner, Ernie M. H. F. Bongers, Mark O'Driscoll, Penny A. Jeggo

**Affiliations:** 1 Double Strand Break Repair Laboratory, Genome Damage and Stability Centre, University of Sussex, Brighton, United Kingdom; 2 Human DNA Damage Response Disorders Group, Genome Damage and Stability Centre, University of Sussex, Brighton, United Kingdom; 3 Department of Human Genetics, Institute for Genetic and Metabolic Disease, Radboud University Nijmegen Medical Centre, Nijmegen, The Netherlands; Washington University School of Medicine, United States of America

## Abstract

Mutations in *ORC1*, *ORC4*, *ORC6*, *CDT1*, and *CDC6,* which encode proteins required for DNA replication origin licensing, cause Meier-Gorlin syndrome (MGS), a disorder conferring microcephaly, primordial dwarfism, underdeveloped ears, and skeletal abnormalities. Mutations in *ATR,* which also functions during replication, can cause Seckel syndrome, a clinically related disorder. These findings suggest that impaired DNA replication could underlie the developmental defects characteristic of these disorders. Here, we show that although origin licensing capacity is impaired in all patient cells with mutations in origin licensing component proteins, this does not correlate with the rate of progression through S phase. Thus, the replicative capacity in MGS patient cells does not correlate with clinical manifestation. However, ORC1-deficient cells from MGS patients and siRNA–mediated depletion of origin licensing proteins also have impaired centrosome and centriole copy number. As a novel and unexpected finding, we show that they also display a striking defect in the rate of formation of primary cilia. We demonstrate that this impacts sonic hedgehog signalling in ORC1-deficient primary fibroblasts. Additionally, reduced growth factor-dependent signaling via primary cilia affects the kinetics of cell cycle progression following cell cycle exit and re-entry, highlighting an unexpected mechanism whereby origin licensing components can influence cell cycle progression. Finally, using a cell-based model, we show that defects in cilia function impair chondroinduction. Our findings raise the possibility that a reduced efficiency in forming cilia could contribute to the clinical features of MGS, particularly the bone development abnormalities, and could provide a new dimension for considering developmental impacts of licensing deficiency.

## Author Summary

Meier-Gorlin syndrome (MGS) is a rare disorder conferring small head circumference, primordial dwarfism, underdeveloped ears, and skeletal abnormalities. Our previous findings suggest that impaired DNA replication could cause the developmental defects in these disorders. Here we expand on those findings by showing that ORC1-deficient cells from MGS patients and depletion of origin licensing proteins also confer impaired centrosome and centriole copy number. Unexpectedly, we show that they also cause a striking defect in the rate of formation and function of primary cilia, hair-like mechano-, and chemo-sensory organelles. Finally we show that defects in cilia function in this context are associated with impaired cartilage formation in a model system. Our findings support the possibility that a reduced efficiency in forming cilia could contribute to the clinical features of MGS, particularly the bone development abnormalities, and could provide a new dimension for considering developmental impacts of licensing deficiency.

## Introduction

Replication in S phase initiates from replication origins, which become “licensed” during G1 phase of the cell cycle [1], [2], [3], [4]. Licensing commences with binding of the origin recognition complex (ORC) followed by recruitment of the pre-replication complex (pre-RC) proteins, CDC6, CDT1 and the MCM2-MCM7 helicase [5]. ORC encompasses six components, ORC1 to 6. ORC2–5 represents the core ORC complex and ORC1 transiently associates with the complex in G1 but dissociates during the transition from G1 to S phase [6]. ORC assembly and origin licensing defines where replication initiates, although only ∼10% of licensed origins are normally utilized for replication [7]. In addition to this essential function, there is increasing recognition that loss of licensing proteins has additional impacts. For example, the ORC subunits contribute to transcriptional gene silencing in yeast and influence heterochromatin formation in Drosophila, mouse and humans [8], [9], [10], [11]. Recently, ORC subunits were shown to associate with chromatin-bound heterochromatin protein 1 (HP1) suggesting that they exert a direct effect on heterochromatinisation rather than the impact being an indirect consequence of impaired licensing [11]. Further, origin licensing proteins localise to centrosomes and siRNA mediated ORC1 depletion causes Cdk2 and cyclin E-dependent centriole and centrosome reduplication [12], [13], [14]. MCM proteins also localise to centrosomes and regulate centrosome copy number [15].

Primary cilia are sensory organelles that grow from a basal body, which represents a modified centriole [16], [17]. Since cilia and centrosome/centriole biogenesis are overlapping and interdependent processes, there is a close relationship between defective centrosome and cilia formation and/or function. For example, pericentrin (PCNT), a core centrosomal protein can, at least in some situations, cause impaired cilia function [18], [19], [20], [21]. Cilia can be either motile (often called flagellae) or immotile, such as primary cilia. Primary cilia are found in most mammalian cell types and function as mechano- and chemosensory organelles by using intraflagellar transport proteins to receive and transduce extracellular signals [22], [23]. Indeed, recent studies have shown the dependence of several signalling pathways on primary cilia, of which a prime example is Hedgehog (Hh) signalling. The binding of the Hh ligand to Patched-1 leads to translocation of Smoothened (Smo) to the ciliary membrane and activation of the Gli1 and Gli2 transcription factors, which play central roles in the Hh pathway [23]. However, other fundamental pathways including Wnt signalling also function via cilia [24]. Importantly, defects in primary cilia formation and/or function are associated with multiple developmental disorders termed “ciliopathies” [17].

Recently, mutations in genes encoding ORC1, ORC4, ORC6, CDT1, and CDC6 were identified in patients displaying Seckel syndrome (SS) and/or Meier-Gorlin syndrome (MGS) [25], [26], [27]. SS, Majewski osteodysplastic primordial dwarfism (MOPD) type II and MGS represent three disorders which share overlapping clinical features that include pronounced microcephaly, severe intrauterine growth retardation and post natal growth delay [28], [29], [30]. Bone abnormalities are also commonly observed in these disorders. However, although there are overlapping phenotypes, each disorder is characterized by distinctive clinical features. For example, MGS is characterized by severely reduced or absent patellae and small/abnormal ears.

The identification of these genetic defects causing profound developmental abnormalities has the potential to provide insight into the underlying developmental processes. In our initial study reporting mutations in *ORC1* in SS/MGS patients, we showed that cell lines derived from ORC1-deficient patients display an impaired ability to sustain rapid replication and argued that this might be causally related to the clinical manifestation [26]. The identification of mutations in *ATR*, which encodes ataxia telangiectasia mutated and Rad3 related (ATR) protein, also functions during replication to maintain replication fork stability, added to the notion that impaired replicative capacity might underlie SS [31]. However, the fact that deficiency in ORC1 also impairs centrosome stability and the close correlation between centrosomal defects and microcephaly, raised the possibility that additional impacts of licensing deficiency might contribute to the clinical features observed in patients [14], [32]. The aim of this study was to examine the broader impact of loss of origin licensing proteins with a consideration of their potential relevance to developmental processes. Extending our initial analysis to cells derived from MGS patients with mutations in additional licensing components, we show that although all cell lines had reduced licensing capacity, there was not a correlation between impaired replicative capacity and clinical manifestations. However, we found that siRNA of licensing components conferred modest defects in centrosome and centriole copy number and organization but importantly we observed marked defects in cilia formation and its consequent signaling function. This represents an important novel pathogenic mechanism potentially underlying the clinical manifestations conferred by deficiency in licensing proteins. We propose that impaired cilia formation represents an important phenotype that should be considered in evaluating the clinical manifestations of MGS, raising the possibility that MGS could be considered as a ciliopathy.

## Results

### Patient cells with mutations in genes encoding origin licensing components have diminished capacity to activate replication origins

**Figure 1:**
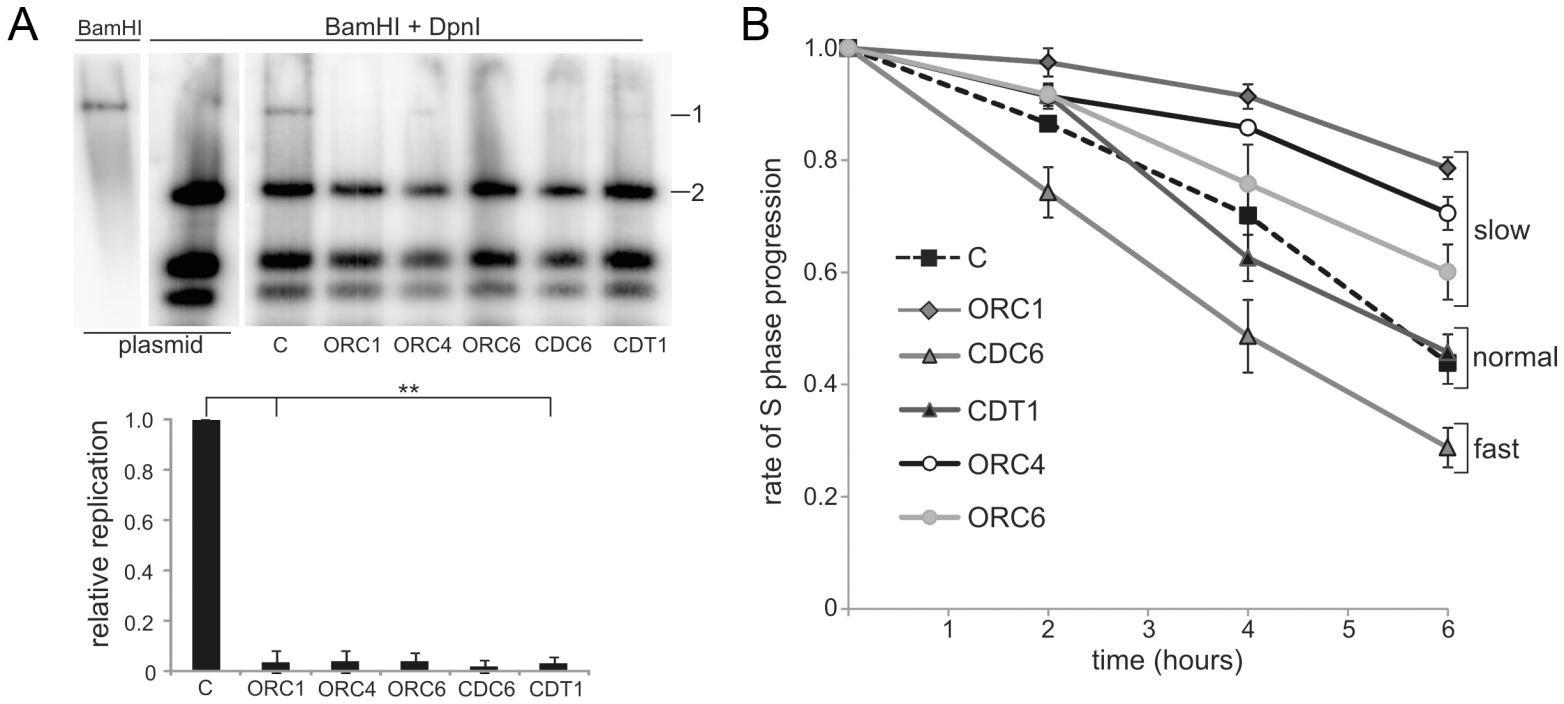
Meier-Gorlin syndrome patients LBLs display impaired origin licensing capacity; some but not all lines show impaired S phase progression. (a) EBV uses virally encoded EBNA-1, oriP and the host cell origin licensing complex for replication. ORC activity was assessed by the replicative capacity of plasmid-294, which encodes OriP and EBNA-1 in a control LBL (C) and LBLs derived from MGS patients with mutations in *ORC1, ORC4, ORC6, CDC6* and *CDT1*. Following transfection of the EBV episome into LBLs and incubation to allow replication, episomal DNA was extracted and examined after *BamH1* or *BamH1+Dpn1* digestion using plasmid-294 as a probe. *Dpn1* degrades unreplicated plasmids that retain bacterial Dam-dependent methylation. The EBV episome has a single *BamH1* site causing linearization after digestion. Although replication of EBV is less efficient in LBLs compared to hTERT immortalised fibroblasts, ∼5% of the EBV plasmids underwent replication in control cells as shown by the presence of full length episomes (band 1) after *Dpn1+BamH1* digestion. The level in MGS patient LBLs is substantially reduced. For quantification, the level of the full length plasmid band (1) was plotted relative to one of the *Dpn1* digestion products (2) and normalised to that obtained in the control (C). Efficient episomal transfection was shown by the similar level of digestion products in all samples. Results represent the mean of two experiments. The reduction was highly significant (t-test, 1-tailed equal variance. Nomenclature used throughout: no significance (ns) P>0.05, * P<0.05, ** P<0.01). (b) Control (C) and ORC1 LBLs were BrdU labelled for 30 min and incubated for the indicated times before fluorescence-activated cell sorting (FACS). The percentage of early S phase cells was assessed. The rate of loss of BrdU^+^ early S phase cells represents the speed of S-phase progression. LBLs with mutations in *ORC1, ORC4* and *ORC6* show an impaired rate of S phase progression; CDT1-deficient LBLs were similar to control LBLs and the CDC6-deficient LBLs progressed more rapidly through S phase.

Cultured lymphoblastoid cell lines (LBLs) derived from MGS patients with mutations in *ORC1*, *ORC4*, *ORC6*, *CDT1* and *CDC6* grow efficiently demonstrating that the mutations do not fully abrogate origin licensing, which is essential for cell growth (the mutations in these cell lines are described in Table S1). Since only ∼10% of licensed origins are utilized during replication, it is likely that even substantially decreased licensing capacity does not grossly impair cell growth [2], [33], [34]. To assess origin licensing capacity, we previously monitored the replication of Epstein-Barr virus (EBV) episomes. EBV uses a viral replication origin (oriP; origin-containing plasmid) with the host cellular ORC machinery and demands a high licensing capacity for efficient replication [35]. Using this assay with patient-derived ORC1-deficient hTERT-immortalised fibroblasts, we previously reported diminished EBV replication compared to control fibroblasts [26]. Since fibroblasts from MGS patients mutated in *ORC4*, *ORC6*, *CDT1,* and *CDC6* were unavailable, we adapted the assay to monitor episome replication in patient-derived LBLs. Following transfection of EBV episomes into control and patient-derived LBLs, the level of replicated episomal DNA was monitored by Southern analysis (Figure 1A). Although episomal replication was less efficient in LBLs compared to hTERT fibroblasts, ∼5% of the EBV plasmids underwent replication in control cells but this was markedly reduced in ORC1, ORC4, ORC6, CDT1, and CDC6-deficient LBLs. Efficient transfection was shown by the similar level of digestion products in all samples. These results strongly suggest that the mutations in origin licensing complex genes found in MGS patients confer a reduced capacity to initiate replication from EBV oriP.

### Slow progression through S phase does not correlate with the clinical phenotype in MGS

Cell lines derived from ORC1-deficient MGS patients progress slowly through S phase, which, we proposed, might represent a consequence of diminished origin firing and the necessity for active replication forks to traverse greater distances [26]. To examine further whether delayed S phase progression might be causally related to the disease phenotype, we examined S phase progression following a 30 min pulse label with bromodeoxyuridine (BrdU) using patient-derived LBLs with mutations in *ORC4*, *ORC6*, *CDT1, CDC6* and *ORC1*, compared to control LBLs (Figure 1B). Although slow S phase progression was observed in LBLs deficient in ORC1 (as observed previously [26]), ORC4- and ORC6-deficient cells, unexpectedly the CDT1-deficient LBLs showed a similar rate to control LBLs. Further, CDC6-deficient LBLs showed more rapid S phase progression. These findings argue that, although impaired origin licensing capacity caused by mutations in *ORC* genes may confer slow S phase progression, diminished licensing capacity does not necessarily correlate with this phenotype. LBLs proliferate relatively rapidly (compared for example to patient fibroblasts). These findings are consistent with previous studies that cells only use a small fraction of licensed origins for normal growth. Although these findings do not rule out the possibility that diminished replicative capacity might impact in specific developmental situations, they demonstrate that the residual licensing capacity can support the relatively rapid replication observed in LBLs. Further, they reveal that impaired S phase progression is not a universal feature of MGS patient cells and does not correlate with the clinical manifestation of MGS. We, therefore, next examined broader consequences of deficient origin licensing.

### Deficiency in origin licensing proteins impacts centrosome and centriole copy number

**Figure 2:**
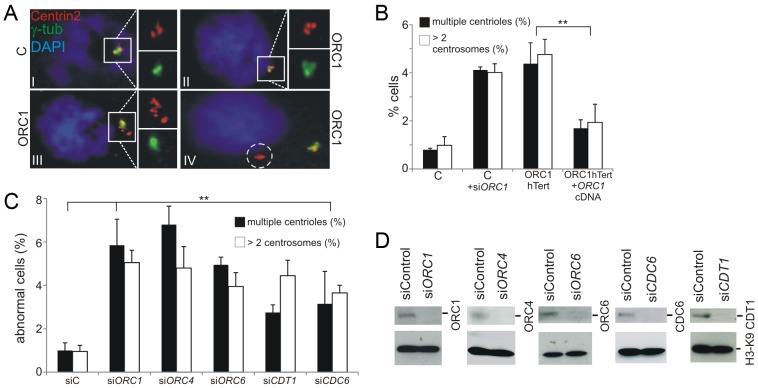
Deficiency in origin licensing proteins results in increased centrosome and centriole copy number. (a–b) Exponentially growing cells were stained with anti-γ-tubulin and anti-Centrin2 to allow visualisation of centrosomes and centrioles, respectively. Cells with >2 centrosomes or >4 centrioles were scored (b). Note that previous studies with ATR-SS cells were carried out using nocodozole to accumulate M phase cells but this analysis was carried out without nocodozole addition to avoid any impact of this drug on spindle assembly^17^. The inset picture (a) shows the types of abnormalities observed. I: normal G2 phase centrosomes and centrioles in control hTERT fibroblasts. ORC1 deficient (ORC1 P4 hTERT) fibroblasts have defects that include II: supernumerary centrosomes and centrioles, III: highly multiple centrioles, IV: centrioles distal from the centrosome. Control-hTERT-immortalised fibroblasts were subjected to ORC1 siRNA and analyzed as above. Analysis was also undertaken in ORC1-deficient hTERT fibroblasts and in ORC1-hTERT fibroblasts following transfection with *ORC1* cDNA. Similar findings were observed using a distinct antibody to mark centrioles (Figure S1). (c–d) Control fibroblasts were treated with the indicated siRNA and examined as in (b) and by Western Blotting to measure knockdown efficiency using the indicated antibodies.

Previous studies have shown that ORC1 localises to centrosomes and that siRNA mediated depletion of ORC1 causes Cdk2 and cyclin E-dependent centriole and centrosome reduplication [12], [13], [14]. In addition, ATR-deficient SS cell lines also show supernumerary centrosomes [14], [31]. Therefore, we examined whether centrosome/centriole abnormalities are also observed in ORC1-deficient patient cells. Using ORC1-P4hTERT fibroblasts, a cell line derived from an ORC1-deficient MGS patient [26], we observed that ∼5% of exponentially growing cells had supernumerary centrosomes and/or displayed distal centrioles (Figure 2A and 2B). Further, this phenotype was observed after siRNA-mediated depletion of ORC1 and was rescued by complementation following transfection of ORC-deficient cells with *ORC1* cDNA (Figure 2B). As a control for this analysis and the subsequent work described below, we also examined primary fibroblasts derived from an MOPD type II patient with mutations in *PCNT*, which encodes pericentrin (PCNT), a centrosomal protein and observed a substantial increase in cells with supernumerary centrosomes consistent with a defect in a core centrosomal protein [36] (Figure S1). Additionally, as controls for the analysis below, we examined primary fibroblasts derived from two Sensenbrenner Syndrome patients, which carry distinct defects in genes encoding primary cilia intraflagellar transport (IFT) proteins, namely *WDR35* (also called *IFT121*) or *IFT43* [37], [38]. Unexpectedly, these cell lines also showed an enhanced fraction of cells with supernumerary centrosomes, although less marked than in the ORC1 or PCNT-deficient lines (Figure S1).

Since the quantification of centrosomes was more difficult to undertake with non-adherent LBLs and since MGS patient fibroblasts deficient in other licensing proteins are not available, we used siRNA to deplete ORC1, ORC4, ORC6, CDT1, and CDC6 in control hTERT fibroblasts and observed a similar frequency of cells with multiple centrosomes (Figure 2C–2D). Thus, impaired centrosome copy number is a general feature of origin licensing deficiency, including loss of pre-RC complex components, and is not specific to loss of ORC1.

### Deficiency in origin licensing proteins ablates the formation of primary cilia in fibroblasts

**Figure 3:**
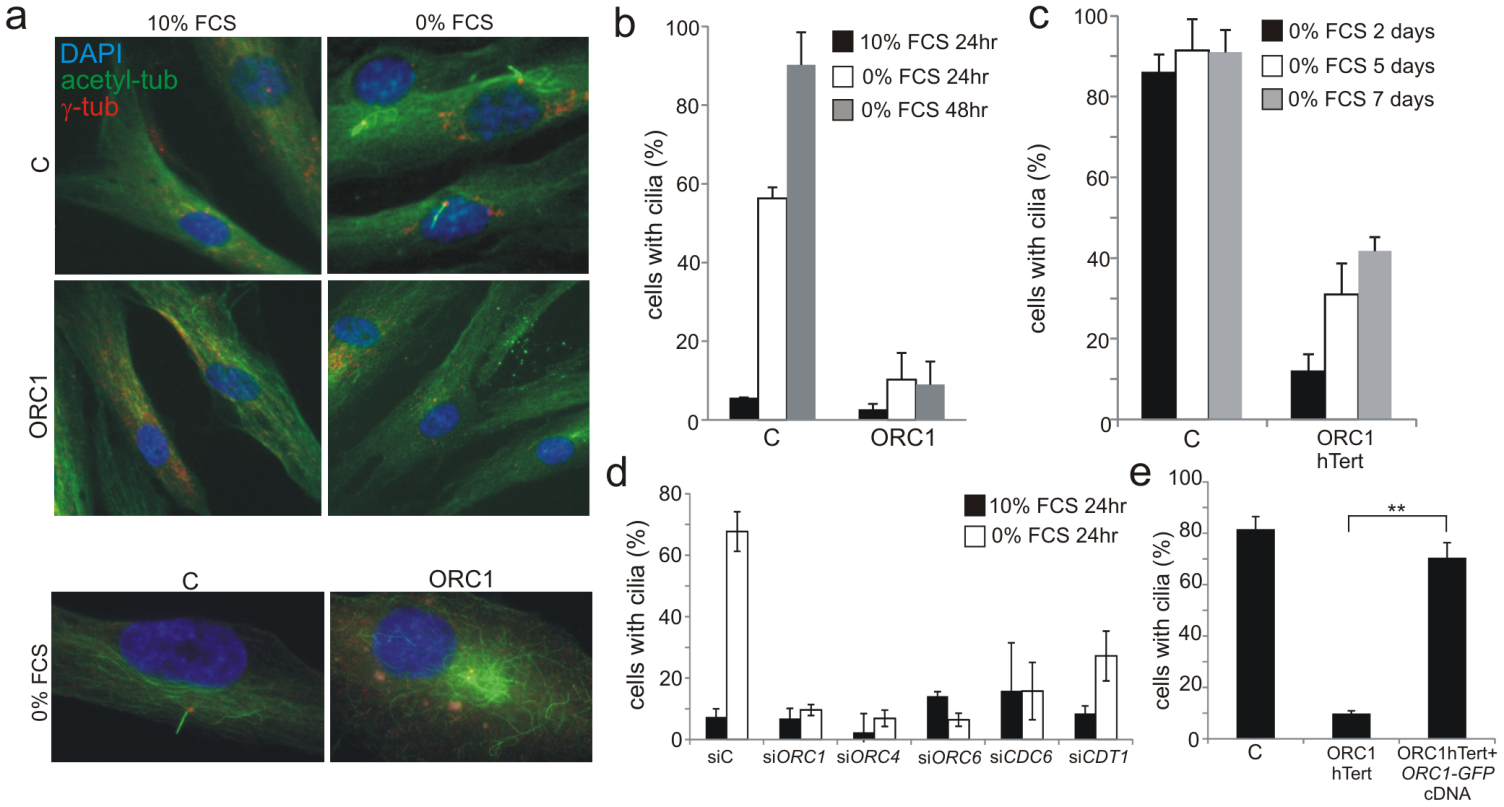
Deficiency in origin licensing proteins dramatically impairs cilia formation. a–b) Control (C) or ORC1-deficient cells were induced to enter G0 by serum starvation for 24 or 48 hr and processed to identify cilia using anti-acetylated tubulin and anti-γ-tubulin antibodies to mark the entire cilia or the basal body, respectively. Lower panel shows that in ORC1-hTERT fibroblasts immunostaining with α-acetylated tubulin reveals extended perinuclear microtubular arrays around the centrosome in distinction to the ordered alignment in control cells and as reported for other cilia defective cells [56]. c) Control (C) or ORC1-deficient hTERT cells were monitored for long term cilia formation as above after the indicated numbers of days of serum depletion. d) Origin licensing proteins were knocked down with siRNA in control hTERT cells, serum starved for 24 hrs then analysed for cilia formation as above. Although a marked defect is observed in cilia formation up to 48 h post serum starvation, cilia can form in around 50% of the cells when examined 4–5 days post serum starvation. e) ORC1-P4 hTERT cells were transfected with empty plasmid or plasmid expressing GFP-tagged *ORC1* cDNA and positive cells detected with anti-GFP antibodies. The percent of GFP^+^ cells, representing those that have been successfully transfected, with cilia was assessed as in panel (d). *ORC1* cDNA expression resulted in rescue of the defect in cilia formation. Figure S2a shows cilia formation in a gfp^+^ versus gfp^−^ cells.

Since cilia develop from centrosomes/centrioles, we next examined whether deficiency in origin licensing proteins affects cilia development. Most mammalian interphase cell-types have a single primary cilium which forms post-cytokinesis in G0/G1 phase and disassembles in two waves spanning the G1/S to G2/M transition [39]. In fibroblasts, cilia can be visualised following cell cycle exit [37], [40]. Initially, we examined cilia formation in hTERT immortalised fibroblasts grown to 70–80% confluency following G0 entry induced by serum starvation. Cilia formed in around 60% and 85% of control fibroblasts at 24 h and 48 h, respectively. Strikingly, there was little detectable cilia formation in ORC1-deficient patient fibroblasts up to 48 h post serum starvation (Figure 3A and 3B). To further test whether this is an absolute defect in cilia formation, control and ORC1-deficient hTERT fibroblasts were serum starved for longer times; cilia formation became more evident at these prolonged times but even after 7 days only 40% of the cells formed cilia (Figure 3C). We used siRNA depletion to examine the requirement for other MGS-associated licensing proteins since we were unable to examine cilia formation in LBLs. Control fibroblasts were subjected to siRNA-mediated knockdown and cultured to examine cilia formation. Strikingly, depletion of ORC1, ORC4, ORC6, CDT1 and CDC6 resulted in dramatically impaired cilia formation similar to ORC1-deficient patient cells (Figure 3D). It is noteworthy that this striking deficiency was observed in the entire cell population although only ∼5% of cells showed abnormalities in centrosome/centriole copy number. Thus, it is unlikely that the impact of ORC1 on ciliogenesis can be a direct consequence of impaired centrosome biogenesis. Expression of GFP-tagged *ORC1* cDNA in ORC1-deficient hTERT fibroblasts fully complemented the defect in cilia formation in GFP-expressing cells detected with anti-GFP antibodies (Figure 3E). To verify that the findings were not due to any impact of the licensing proteins on the ability to enter G0/G1 phase, we monitored the number of G2, mitotic, active G1 and S phase cells in control, ORC1-deficient hTERT fibroblasts and following all siRNA treatments and observed a similar rate of cell cycle exit under all conditions (Figure S3).

In summary, these findings provide strong evidence that loss of origin licensing proteins substantially delays, although does not fully ablate, the ability to form primary cilia.

### ORC1-deficient patient cells show impaired sonic hedgehog signalling

**Figure 4:**
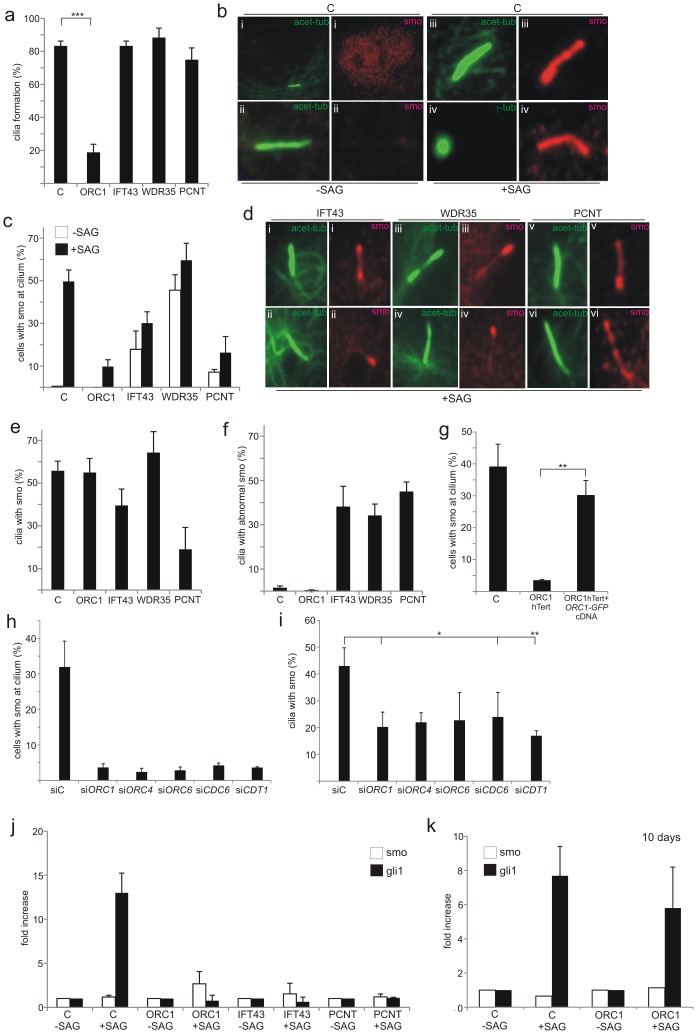
Recruitment of Smo to cilia is deficient in ORC1-deficient fibroblasts. a–f) Primary fibroblasts with the indicated deficiency (ORC1, IFT43, WDR35 or PCNT) were induced to enter G0 phase following serum depletion for 48 hr. The Shh pathway agonist SAG was then added for a further 24 hr. a) shows the percentage of cells with cilia after 3 days serum starvation. b) In control cells in the absence of SAG Smo is localised diffusely in the nucleus but not at the cilium. In the presence of SAG a strong uniform Smo staining is visible along the length of the cilium marked with acetylated-tubulin or extending from the basal body marked by γ-tubulin. c) Shows the percentage of cells with cilia and co-localised Smo with or without SAG treatment. d) Examples of abnormal Smo localisation at the cilium: (i) accumulation at distal tip, basal body and weakly along shaft, (ii) localised to distal tip only, (iii) localized to distal tip and partially along cilium shaft, (iv) localised to distal tip only, (v) accumulation at basal body, (vi) non-uniform distribution along length of cilium. e) Shows the percentage of ciliated cells with co-localised Smo. f) shows the percentage of ciliated cells with abnormally localised Smo. g) the percentage of cells with Smo localisation at the cilia following transfection of ORC1-hTERT cells with GFP-tagged*ORC1* cDNA. Positive GFP cells were detected with anti-GFP antibodies. Smo localisation is quantified in GFP^+^ cells. Images of Smo localisation in Gfp^+^ cells is shown in Figure S2. h and i) percentage of cells with Smo at cilium (h) or percentage of ciliated cells with co-localised Smo (i) following the indicated siRNA. j and k) qRT-PCR analysis of *Gli1* transcript levels in the indicated cells with or without SAG for 24 h (j) or (k) following 7 days serum starvation.

Primary cilia function in many different organs to coordinate and transduce signals, including Sonic hedgehog (Shh) and Wnt-regulated pathway signalling, since they are enriched for specific receptors [16], [41]. Since Shh signalling plays a major role in many developmental processes and since its activation can be monitored in primary fibroblasts, we evaluated whether ORC1 deficiency impacts upon Shh signalling. Cellular responses to secreted Shh ligand are mediated by two trans-membrane proteins, Patched-1 receptor (Ptch-1) and Smoothened (Smo), a pseudo-G protein coupled receptor. Shh ligand binds initially to Ptch-1, which alleviates its suppression of Smo. Smo activation triggers translocation of Gli2 to the nucleus where it regulates the transcription of Shh-pathway response genes, including *Gli1, Ptch1* and *Hhip.* SAG is a chlorobenzeothiophene-containing Shh pathway agonist that functions downstream of Ptch-1 by binding directly to Smo. Treatment with SAG, therefore, causes accumulation of Smo at the cilia. To assess whether the diminished ability to form cilia following loss of ORC1 affects Shh signalling, we examined Smo localisation at cilia following treatment with SAG. We utilised patient derived primary fibroblasts to allow the inclusion of IFT43-, WDR35-, and PCNT-deficient primary fibroblasts as controls. At 72 h post serum starvation in the absence of SAG, we observed that the majority (>80%) of control fibroblasts have formed cilia (Figure 4A; detected using acetylated-tubulin antibodies) but Smo was localised in a diffuse pan nuclear manner (Figure 4B i). When SAG was added for the final 24 h, Smo localised to the cilia, with ∼50% of the cells showing colocalised acetylated-tubulin and Smo (Figure 4B and 4C). IFT43-, WDR35- and PCNT-deficient fibroblasts formed cilia at a similar level to wild-type control cells after serum starvation, although the acetylated-tubulin staining pattern was frequently abnormal (Figure 4D). This is consistent with previous findings that cilia form at close to normal levels in IFT43- and WDR35-deficient patient cells [37], [38]. Although one study has shown that PCNT is required for ciliogenesis, subsequent work using a hypomorphic mouse strain suggested that PCNT was essential for olfactory cilia assembly but dispensable for ciliogenesis in non-neuronal epithelial cells [19], [20]. The normal level of cilia formation here may reflect the latter finding or the fact that PCNT function is not fully abrogated in the patient cells. In contrast to wild-type control cells, Smo localised at the cilia in a detectable fraction of non-SAG treated cells in the three patient-derived cell lines suggesting some potential functional deficiency (Figure 4C). In the presence of SAG, the fraction of cells with Smo localised at the cilia increased slightly but for IFT43- and more markedly PCNT-deficient cells, the fraction with co- localised Smo remained below the level in control cells (Figure 4C). Additionally, in these three cell lines (IFT43, WDR35 and PCNT) the staining for Smo appeared non-uniform compared to that observed in control cells exposed to SAG (Figure 4D). These findings are consistent with the impact of IFT43 and WDR35 on retrograde intraflagellar protein transport (but normal anterograde transport), a downstream step in cilia function, rather than on cilia formation or Smo activation. PCNT deficiency in fibroblasts confers a distinct phenotype with most cells forming cilia normally but with diminished or abnormally co-localised Smo without or with SAG. This substantiates the findings described above that cilia formation is only modestly compromised by PCNT deficiency and demonstrates that cilia function is more markedly impaired.

Finally, ORC1-deficiency results in dramatically impaired cilia formation (as described above) and hence few cells have localised or accumulated Smo either without or with SAG treatment (Figure 4A and 4C). However, assessment of the fraction of ciliated cells that showed co-localised Smo after SAG revealed that ORC1 deficiency, whilst compromising cilia formation, did not affect the ability to localise Smo in the reduced number of ciliated cells (Figure 4E). IFT43, WDR35 or PCNT deficiency conferred a distinct phenotype with only a modest impact on cilia formation but clearly aberrant Smo localisation (Figure 4F). This strongly suggested that ORC1-deficiency dramatically impairs cilia formation (at 72 h post serum starvation) but the function of the cilia that do form is normal for ability to localise Smo. PCNT-deficient cells, however, show a distinct phenotype with only around a quarter of the ciliated cells showing accumulated Smo, providing further insight into the impact of PCNT deficiency on cilia function. Transfection of GFP-tagged *ORC1* cDNA into ORC1-deficient hTERT fibroblasts complemented the lack of Smo localisation in GFP-expressing cells detected with anti-GFP antibodies (Figure 4G). A failure to localise Smo at the cilia was also observed following siRNA of ORC1, ORC4, ORC6, CDC6 and CDT1 (Figure 4H), mainly due to the greatly reduced cilia formation. In the cells that did form cilia Smo localisation was detectable, although somewhat reduced (Figure 4I).

Shh signalling results in the transcriptional up-regulation of *Gli1*, providing a further assay to monitor cilia function. Using quantitative Real Time-PCR (q-RT-PCR) we assessed the change in *Gli1* transcript levels in control, ORC1, PCNT or IFT43 patient cells either without or with SAG treatment. Control cells showed a greater than tenfold increase in *Gli1* transcript levels after SAG but no change was observed in the patient cells examined (Figure 4J). Finally, since 40% of ORC1-hTERT cells formed cilia at prolonged times (7 days) post serum starvation, we examined whether this correlated with functional Shh signalling assessed by *Gli1* transcript levels. Indeed, at 10 days post serum starvation, *Gli1* levels were substantially increased suggesting that the cilia that form at prolonged times in ORC1-hTERT cells are functional for Shh signalling (Figure 4K).

We conclude that an impaired ability to form cilia caused by ORC1-deficiency impacts upon Shh signalling. However, the impaired response is a consequence of diminished cilia formation rather than function. In contrast, IFT43- and PCNT-deficient fibroblasts show altered or impaired cilia function although the ability to form cilia is not dramatically impaired.

### Diminished ORC1 impairs the cilia-dependent response to PDGF

**Figure 5:**
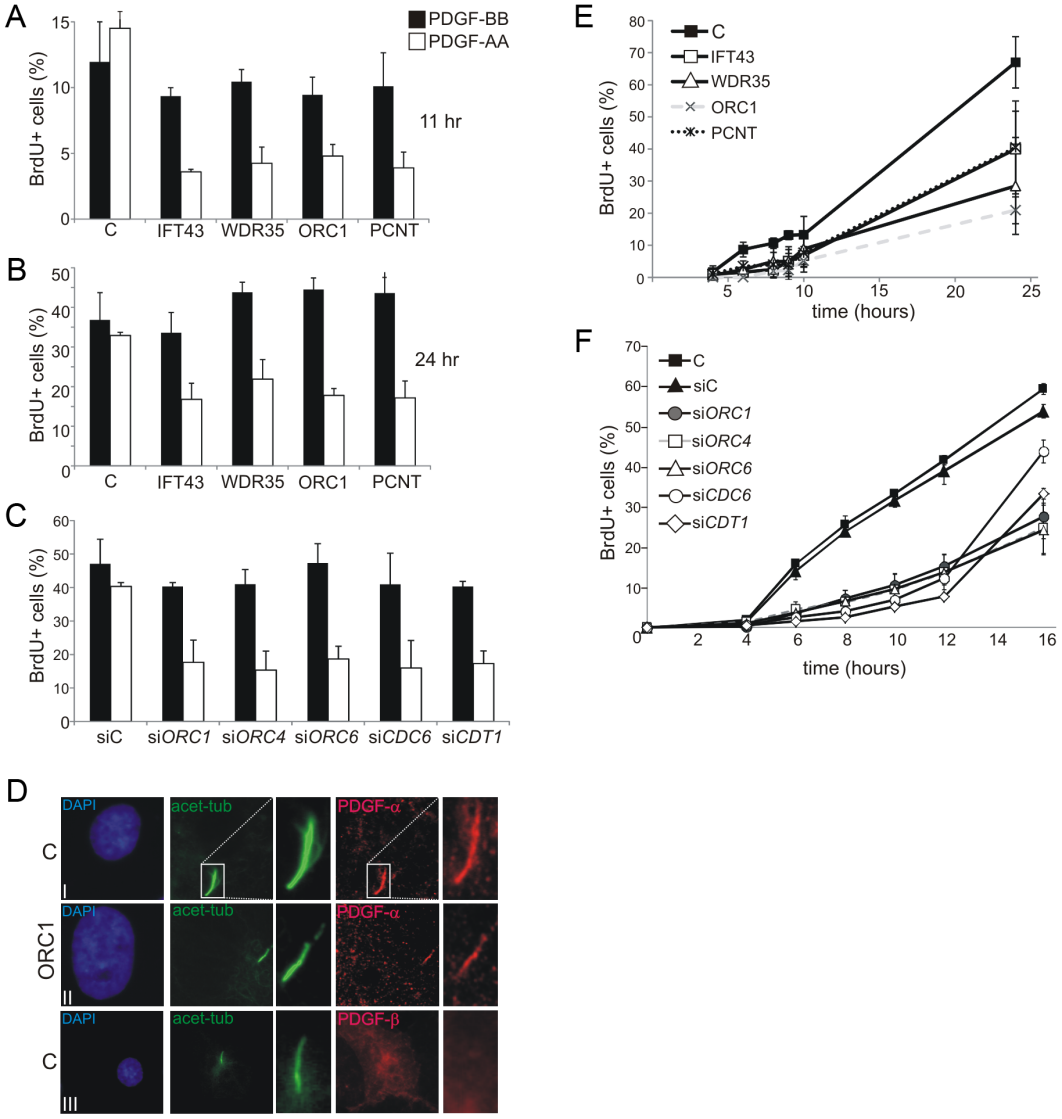
Deficiency in origin licensing proteins impairs cilia function in response to platelet-derived growth factor (PDGF). a–b) Fibroblasts were induced to enter G0 phase following serum depletion for 48 hr. PDGF-AA or –BB and BrdU was then added and the percentage of S phase cells, monitored as BrdU^+^ cells, was estimated by immunofluorescence 11 (a) and 24 hr (b) later. The receptor for PDGF-AA is located in cilia whilst the –BB receptor is on the cell membrane. c) Analysis as in a) following the indicated siRNA. d) Cellular localisation of PDGFR-α or β. Anti-PDGFR-α or –β antibodies were used to examine the localisation of the two PDGF isoforms in control (C) or ORC1-P4 hTERT cells. PDGFR-α localised to the cilia, identified using anti-acetylated tubulin in control and ORC1-P4 cells although fewer cilia formed in the latter cells. PDGFR-β showed pan cellular localisation but did not co-localise with the cilium. e) Cells were induced to enter G0 phase following serum depletion for 48 hr. Serum was then re-added and the fraction of BrdU^+^ S phase cells monitored at the indicated times. The top panel shows the results with a control (C) primary fibroblasts, 48BR, primary fibroblasts from Sensenbrenner syndrome patients (*IFT43*-mutated and *WDR35*-mutated), PCNT defective fibroblasts and an ORC1 deficient line MGS cells. Both Sensenbrenner syndrome lines and PCNT cells showed a delayed S phase entry, similar to ORC1 defective MGS, compared to the control primary line. f) Analysis of a control hTERT immortalised cell line either without knockdown (C), treatment with control siRNA oligonucleotides (siC) or with oligonucleotides specific (si) for *ORC1, ORC4, ORC6, CDC6* or *CDT1*. Knockdown efficiency was assessed and was similar to that observed in Figure 2. Note that the control hTERT immortalised line (C) enters S phase more rapidly that the primary fibroblasts line making it difficult to allow a direct comparison between the S phase entry kinetic defects observed in the Sensenbrenner syndrome primary lines in (e).

The cellular response to a specific isoform of platelet-derived growth factor (PDGF), which is recognised by a receptor located in cilia, represents another cilia-dependent response which links to cell cycle entry and subsequent DNA replication [42]. Two major PDGF ligand isoforms and their corresponding receptors have been identified. PDGF receptor α (PDGFRα) specifically localises to primary cilia, is upregulated in serum-starved cells, and responds to the PDGF-AA ligand isoform [43]. In contrast, the PDGFRβ receptor, which responds to the PDGF-BB isoform, localises predominantly on the cell membrane. A primary role of PDGF signalling is to promote cell cycle entry from G0 [44]. We exploited PDGF-AA and –BB to examine cilia function following cell cycle exit and re-entry. This system was exploited since it allows the impact of ORC1 deficiency on membrane dependent versus cilia dependent signalling to be assessed. Following growth to 70–80% confluency and serum starvation for 48 h (conditions promoting cilia formation), cells were treated with PDGF-AA or BB isoforms for 11 or 24 h. Cell cycle re-entry was monitored as the percentage of BrdU positive (BrdU^+^) cells by immunofluorescence (IF). Whilst control fibroblasts showed a similar ratio of BrdU^+^ cells when exposed to PDGF-AA or -BB, ORC1-deficient fibroblasts showed substantially diminished BrdU^+^ cells following PDGF-AA addition (Figure 5A and 5B). A similar result was observed in cells deficient in IFT43, WDR35 or PCNT consistent with the known role of these proteins in cilia protein transport (IFT43 or WDR35) or cilia function (PCNT), as demonstrated above. Examination of ORC1 siRNA in control fibroblasts demonstrated a similarly impaired response to PDGF-AA (Figure 5C). Furthermore, siRNA mediated depletion of ORC4, ORC6, CDT1 or CDC6 similarly diminished the response to PDFG-AA without impact on the PDGF-BB response (Figure 5C). Finally, we examined the cellular localisation of PDGFR-α and PDGFR-β confirming that PDGFR-α localises to cilia whilst PDGFR-β showed a pan-cellular localisation (Figure 5D). Notably, PDGFR-α localised to the few cilia that formed in ORC1-deficient cells, consistent with the notion that these cilia were functionally normal.

Collectively, these data demonstrate that the defect in cilia formation caused by depletion of origin licensing proteins impacts upon the cilia-dependent response to growth signals.

### Impaired cilia formation also causes delayed S phase entry following cell cycle exit and re-entry by serum addition

Previously, we observed that ORC1-deficient fibroblasts show delayed S phase entry after cell cycle exit and re-entry following serum addition [26]. We concluded that this phenotype could be due to a ‘licensing checkpoint’ that precludes S phase entry until a critical level of origin licensing in G1 is achieved [26], [45], [46]. However, we noted that the assay involved conditions that corresponded to those described above for monitoring cilia function except that serum was employed to promote cell cycle entry rather than PDGF isoforms. We, therefore, considered it possible that our previous findings might predominantly reflect impaired ciliogenesis and/or ciliary function rather than a ‘licensing checkpoint’. To examine this, we monitored S phase entry using BrdU labelling following cell cycle exit and re-entry after serum re-addition in IFT43-, WDR35-, or PCNT-defective fibroblasts. Strikingly, all three lines showed delayed S phase entry compared to control fibroblasts, a phenotype similar to that observed in ORC1-deficient cells (Figure 5E). Next, we used siRNA-mediated depletion in control fibroblasts to examine the requirement for additional origin licensing components. Strikingly, whereas fibroblasts treated with control siRNA commenced S phase entry within 4–6 hrs following serum addition, entry was delayed in cells subjected to siRNA of MGS-associated licensing proteins (Figure 5F). Interestingly, in this assay the defect was less marked following cell cycle exit at 7 days post serum starvation, consistent with the notion that functional cilia can form in this context after prolonged times in the ORC1 deficient cells (Figure S4). These findings provide strong evidence that this assay monitors cilia function in response to growth factors. Although the contribution of a licensing checkpoint cannot be eliminated and, indeed, the two mechanisms are not mutually exclusive, the data obtained with the IFT43 WDR35 and PCNT-defective fibroblasts demonstrate that cilia dysfunction can significantly impair cell cycle progression.

### ORC1-deficient patient cells are impaired in an *in vitro* model system for chondrogenesis

**Figure 6:**
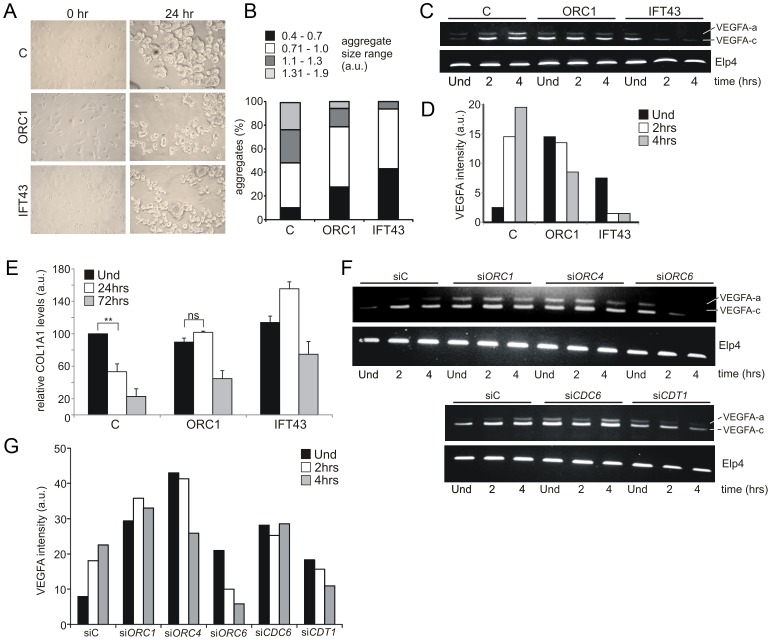
ORC1 Meier-Gorlin syndrome and IFT43 Sensenbrenner syndrome fibroblasts exhibit impaired chondroinduction. a–b) Phase contrast images (40×) of control (C) hTERT, ORC1-deficient MGS (ORC1-P4hTert) and *IFT43*-mutated Sensenbrenner (IFT43) patient derived fibroblasts at 0 hr and 24 hr following addition to aggrecan coated plates. Size distribution of aggregates from control (C), ORC1 and IFT43 fibroblasts following 24 hr micromass culture in aggrecan coated plates (n = 350 aggregates scored per line). Larger aggregate size was a feature of control fibroblasts following chondroinduction compared to ORC1 and IFT43 cells. c–d) Semi-quantitative RT-PCR amplification of *VEGFA* isoform a (upper band) and isoform c (lower band) either uninduced (Und) or during chondroinduction. Both isoforms were induced in control fibroblasts (C) upon chondroinduction. Whilst IFT43 cells exhibited higher endogenous levels of *VEGFA* isoform c, it was not maintained upon chondroinduction. Isoform a also was not induced after chrondroinduction. Similar findings were observed for ORC1 cells, although the high endogenous level of isoform c reduced less dramatically than that in IFT43 cells but did not increase in as in control cells. *ELP4* was used as an amplification control. Panel (d) shows the combined quantification for isoforms a and c from the above panel. Similar findings have been observed in three independent experiments. e) Type I collagen represents a negative marker for chondroinduction as its levels decrease as differentiated chondrocytes secrete a specific extracellular matrix. Consistent with this, *COL1A1* levels, as monitored by quantitative RT-PCR were found to decrease in control fibroblasts (C) during chondroinduction. Interestingly, both ORC1 and IFT43 defective patient derived cells exhibited similar levels of endogenous *COL1A1* compared to control but by 48 h, the levels had less dramatically diminished compared to control cells. The results represent the mean of three experiments. f–g) Analysis of a control hTERT cell line treated with control siRNA oligonucleotides (siC) or with oligonucleotides specific (si) for *ORC1, 4, 6, CDC6* or *CDT1*. Cells were uninduced (Und) or induced on a chondrogenic matrix then assayed for *VEGFA* expression as detailed in (c–d). Panel (g) shows the combined quantification for isoforms a and c from the above panel. Similar findings have been observed in two independent experiments.

MGS patients display pronounced cartilage and bone defects, including markedly small ears, small or absent patella, micrognathia, delayed bone age, and short slender ribs. Coupled with the established role of cilia in chondrogenesis, we examined the chondrogenic potential of ORC1-deficient MGS cells [47], [48]. A model system for chondroinduction using fibroblasts, which share a common mesenchymal origin with chondroctyes, necessitates cell cycle exit and subsequent association of single cells into aggregates upon exposure to a chrondrogenic matrix [49], [50]. The size distribution of aggregates formed in ORC1-defective and IFT43-defective fibroblasts was smaller than those formed in control fibroblasts (Figure 6A–6B). Vascular Endothelial Growth Factor A (*VEGFA*) is induced during chondroinduction and chondrogenesis. Using semi-quantitative RT-PCR, control fibroblasts showed enhanced levels of two *VEGFA* transcript isoforms following culture upon the chondrogenic matrix (Figure 6C and 6D). Both ORC1-defective and IFT43-defective fibroblasts exhibited enhanced endogenous levels of the smaller isoform (isoform c), which diminished rather than increased upon chondroinduction (Figure 6C and 6D). The larger VEGF isoform (a) similarly increased in control but not in ORC1-defective or IFT43-defective cells following chondroinduction. In converse to *VEGFA*, type 1 collagen (*COL1A1*) is normally transcriptionally down regulated during chondroinduction (Figure 6E). Using qRT-PCR to monitor *COL1A1* transcript levels, we observed that they were high in control fibroblasts, decreased at 24 h following culture upon the chondrogenic matrix and reduced to one fifth of the level in uninduced cells by 72 h; in contrast, in ORC1- and IFT43-deficient fibroblasts the *COL1A1* levels were not decreased at 24 h and less substantially decreased at 72 h (2 to 2.5 fold decreased for ORC1-deficient cells (Figure 6E). Changes in *VEFGA* transcript levels were also examined in control hTERT cells following siRNA knockdown of the other MGS-associated origin licensing proteins, including ORC1 (Figure 6F). The results obtained following transfection with control oligonucleotides were similar to those shown for control hTERT cells (Figure 6C and 6F) showing an increase in *VEGFA* transcript isoforms following culture upon the chondrogenic matrix. In contrast siRNA mediated knockdown of ORC1, ORC4, ORC6, CDT1 or CDC6 resulted in high endogenous levels of *VEGFA* with either no change or a decrease after chondroinduction, which resembled the response seen in the patient cells. (Figure 6C, 6F and 6G).

Collectively, this analysis using an established model culture system for chondrogenesis with IFT43-defective cells provides evidence that chondroinduction requires cilia function. Whilst ORC1-defective fibroblasts show a milder defect, their response to chondrogenic matrix is clearly abnormal. Furthemore, siRNA mediated silencing of the other MGS genes, *ORC4, ORC6, CDT1* or *CDC6* was also clearly associated with an aberrant chondroinduction phenotype. Together, this highlights a novel link between defects in pre-RC components and programmed differentiation of clinical relevance to chondrogenesis in MGS.

## Discussion

Defects in origin licensing proteins confer MGS (and in some instances SS), which is characterised by a range of clinical features including severe microcephaly, small ears, small/absent patellae, and defects in bone development [25], [26]. Origin licensing proteins have a canonical function in licensing replication origins during G1 for replication in S phase [1], [2], [3], [4]. Taken together with the fact that mutations in *ATR*, which functions to maintain replication in the face of DNA damage, also cause SS, this raised the possibility that the clinical features might be a direct consequence of insufficient replicative capacity. Previously, we observed that ORC1-deficiency caused slow progression through the S and G1 phase and proposed that a failure to sustain rapid replication during critical developmental stages might underlie the clinical manifestations [26]. Here, we show that cells derived from MGS patients with defects in additional licensing components (ORC4, ORC6, CDT1 and CDC6) have diminished origin licensing capacity. However, although slow S phase progression was observed in some lines, it was not a consistent phenotype. Coupled with the fact that such patient LBLs grow efficiently (and LBLs are rapidly growing cells), this suggests that diminished licensing capacity in MGS does not dramatically impede cell growth even under rapidly growing conditions and does not correlate with clinical phenotype. Since only ∼10% of licensed origins are utilised during normal replication, it is likely that efficient replication can pursue even with markedly reduced licensing capacity. Although we cannot eliminate the possibility that impaired replicative capacity might contribute in some cell types to the disease phenotype, we examined the consequences of additional impacts of origin licensing deficiency.

Extending our findings following siRNA-mediated silencing of ORC1, we show that loss of additional licensing proteins (ORC4, ORC6, CDT1, and CDC6) also confer a subtle defect in centrosome and centriole copy number [14]. Further, as a novel and unexpected finding, we demonstrate that such defects dramatically impact upon cilia formation. Although only a subfraction of cells depleted for licensing proteins have supernumerary centrosomes/centrioles, there is a marked defect in cilia formation affecting the entire population. Indeed, cilia failed to form in some cells where centrosome numbers appeared normal (data not shown). Thus, the defect in ciliogenesis in patient cells cannot be a direct consequence of defective centrosome biogenesis. Previous studies have suggested that ORC1 regulates centriole and centrosome copy number via interactions with Cyclin E [14]. Additionally, ORC1 is localised to centrosomes via a process involving Cyclin A. However, it is unclear how such a model would exert a major impact upon cilia formation. Thus, these two phenotypes (the impact on centrosomes versus cilia) may be the consequence of distinct aspects of deficiency in origin licensing proteins and it is currently difficult to disentangle whether defective cilia arise as a consequence of a direct role of origin licensing proteins in cilia formation or are a downstream consequence of dysfunctional centrosome/centriole organisation. Although ORC1 localises to centrosomes in control cells, we have not yet been able to assess whether there is any lack of function or malfunction of ORC1 at centrosomes in patient cells. Unexpectedly, we observed that Sensenbrenner syndrome cells, which have a known defect in intraflagellar transport, also display impaired centrosome and centriole stability (Figure S1). Thus, the connection between cilia, centrosomes and centrioles is complex and multiple proteins are likely required for their efficient biogenesis. We demonstrate that depletion of origin licensing components does not affect the kinetics of cell cycle exit upon serum starvation making it unlikely that the findings can be explained by an impaired ability to exit the cell cycle (Figure S3). Importantly, however, cilia do form in licensing deficient cells but do so substantially more slowly. Interestingly, pre-replication complex formation and ciliogenesis both occur during G0/G1 phase and it is possible that signalling via interactions with Cyclin A or E delays appropriate signals to initiate the latter processes.

An important consideration is whether these novel and unexpected consequences of deficiency in origin licensing proteins contribute to the clinical features of MGS. As one step towards evaluating this, we exploited a cell based model for chondrogenesis. Although this assay involves the differentiation of fibroblast cells into chrondrocyte-like cells and, thus, may not fully represent the *in vivo* differentiation process, chrondrogenesis *in vivo* necessitates a similar process involving cell cycle exit and response to differentiation factors. Importantly, this model system allows use of patient derived material. Strikingly, we show that this differentiation process is defective in IFT43-defective Sensenbrenner syndrome cells, which are impaired in intraflagellar transport providing strong evidence that the differentiation step involves cilia-dependent signalling. Importantly, we observe that ORC1 deficient patient cells and siRNA mediated silencing of the other pre-RC MGS genes (*ORC4, ORC6, CDT1* and *CDC6*) in control fibroblasts also exhibit specific impairments in this assay. These findings provide a further demonstration that licensing proteins impact upon cilia function and yield potential novel insight into how deficiency in origin licensing proteins might impact upon skeletogenesis.

Microcephaly represents a further clinical characteristic of MGS/SS. Significantly, several genetic defects that cause primary microcephaly represent centrosomal proteins. Moreover, PCNT, which is mutated in MOPD II, is a centrosomal protein with a characterised role in ciliogenesis [19], [20]. Significantly, we show here that PCNT-deficient patient derived cells also display a defect in cilia function. There is strong evidence that microcephaly can arise from a failure to efficiently expand the pool of neuronal progenitor cells via a process that necessitates a timely switch from asymmetric to symmetric cell division [32]. The centrosome is critical in promoting this switch through regulation of the orientation of the cleavage plane furrow. It is possible that cilia function is also required during this early stage of neurogenesis. Moreover, Shh signalling also has an important role during neurogenesis and disruption of cilia function leads to cerebellar defects [51], [52], [53].

Collectively, our studies raise the possibility that MGS should be considered as a ciliopathy. However, some of the clinical features of MGS are distinct to other ciliopathies [16], [54], [55]. For example, kidney dysfunction is frequently observed in ciliopathy disorders and has rarely been reported in MGS. Whilst abnormalities in brain superstructure are frequently observed in ciliopathies, microcephaly is not a consistent feature and is not a feature of Sensenbrenner syndrome. However, skeletal defects are commonly seen in ciliopathies, as typified by Sensenbrenner syndrome. In assessing this, it is important to appreciate that the cilia defect caused by origin licensing deficiency is not absolute and cilia can form albeit substantially slower than in control cells. Moreover, the cilia that form, in contrast to those arising in Sensenbrenner syndrome or PCNT deficient cells, appear to be functionally normal. Thus, it is likely that the impact of impaired cilia formation may depend upon cell type; in those situations where rapid signalling is required, such as during neurogenesis, the impact of delayed cilia formation could be significant whilst in other tissues, such as kidney, where ciliated cells may be long lived, the impact might be less consequential. Finally, other aspects of licensing deficiency may also contribute to the clinical manifestations. Subtle differences in function could, when combined, have profound clinical manifestations making it difficult to untangle linear relationships. Nonetheless, the defect in timing of cilia formation reported here is striking and, as we show for PDGF signalling, can contribute to altered DNA replication kinetics. Thus, our findings represent a novel dimension to the consideration of the developmental impact of pre-RC licensing component deficiency.

In summary, we report here that defects in multiple licensing proteins that arise in MGS patients cause modest defects in centrosome and centriole copy number but marked defects in the rate of cilia formation and consequently cilia function. We provide novel examples of how signalling via cilia can affect cultured cells including impacts on sonic hedgehog signalling, PDGF-mediated cell cycle progression, and chondroinduction.

## Materials and Methods

### Cell culture

LBLs utilized are control (GM2188), deficient in ORC1 (ORC1-P1/CV1759), ORC4 (GM018380), ORC6 (GM020744), CDT1 (GM020792) and CDC6 (GM013107) Mutations are given in [25], [26]. LBLs were grown in RPMI medium supplemented with 15% foetal calf serum (FCS), penicillin, and streptomycin. Primary human fibroblasts utilized were control (1BR), Orc1-deficient (Orc1-P4) [26], IFT43 (CL10-00031) [37] WDR35 (CL10-00021) [38] and PCNT (ASB). hTERT derivative fibroblasts were control (1BR3hTERT or 48BRhTERT) and Orc1 (ORC1-P4hTERT). Fibroblasts were grown in MEM with 15–10% FCS, 1% non-essential amino acids (NEAA) and 1% antibiotics. ORC1, ORC4, ORC6, CDT1, CDC6 and control siRNA was carried out using the appropriate Smartpool (Dharmacon, Lafayette, Colorado) and Metafectene Transfection Reagent (Biontex, Munich, Germany).

### Immunofluorescence

Cells grown on coverslips were fixed with 3% formaldehyde for 10 min and permeabilized in 0.5% Triton-X100. For BrdU staining, DNA was denatured in 2 N HCl for 30 min. After antibody treatment and staining with 4,6-diamidino-2-phenylindole (DAPI), coverslips were mounted in Vectashield mounting medium (Vector Laboratories, Burlingame, California). Samples were incubated with primary antibodies for BrdU (BU20A), CenPF, CPAP, phospho-H3 (Santa Cruz, Santa Cruz, California), Centrin 2 (a kind gift from Dr E. Scheibel), γ-tubulin, acetylated-tubulin (Sigma, St. Louis, Missouri), phospho-Rb (Cell Signaling, Beverley, Massachusetts), anti-GFP (Invitrogen) and Smoothened (Abcam, Cambridge UK). Secondary antibodies were from Sigma.

### S phase progression assay

BrdU-labelled cells were fixed in 70% ethanol (-20°C), treated with 2 M HCl in PBS for 20 min, washed in PBS/1% FCS, incubated in 0.1 M Na-tetraborate for 2 min, re-washed in PBS/1% FCS and incubated with FITC-conjugated monoclonal anti-BrdU antibody solution (Santa Cruz, Santa Cruz, California). Finally, cells were stained with 10 µg/ml propidium iodide and 0.5 mg/ml RNase in PBS for 30 min. Analysis was performed on a FACScan (Becton Dickinson, Franklin Lakes, New Jersey) or a FC500 (Beckmann Coulter, Indianapolis, Indiana). Identification of cell compartments was as previously described [26].

### Cilia formation assay

Fibroblasts were grown to 70–80% confluency followed by serum starvation in MEM containing 0.5% (primary cells) or 0.1% (hTERT immortalized cells) FCS for 1–7 days to promote entry into G0. Cells were processed for immunofluorescence as above and cilia visualized with anti-acetylated tubulin and γ-tubulin antibodies.

### Shh pathway assay

Fibroblasts were serum starved for 2–3 days in MEM containing 0.1% FCS. Then MEM with or without 1 µM SAG (Smoothened agonist, Calbiochem, Billerica, Massachusetts) was added for a further 24 hrs. Cells were processed for immunofluorescence as above. Cilia or the basal body were identified by antibodies against acetylated-tubulin and γ-tubulin then Smoothened staining at the cilium assessed.

### Cilia function assay

Fibroblasts were serum starved for 2–3 days in MEM containing 0.5% (primary cells) or 0.1% (hTERT cells) FCS. Then MEM with FCS or with 50 ng/ml PDGF-AA, PDGF-AB or PDGF-BB (Sigma, St. Louis, Missouri) was added. S-phase cells were identified by labeling with 10 µM BrdU (Becton Dickinson, Franklin Lakes, New Jersey) and processed for immunofluorescence as above.

### Centrosome analysis

Cycling fibroblasts were processed for IF as above and centrosomes or centrioles visualized with anti-γ-tubulin and anti-Centrin-2 antibodies, respectively.

### EBV origin licensing assay

1×10^7^ cells were transfected with 10 µg OriP and EBNA-containing plasmid p294 using Calcium Phosphate. Plasmid DNA was isolated after one population doubling using a modified Hirt extraction procedure. Plasmid DNA was linearised with *BamHI* alone or together with *DpnI*. DNA was repurified with a Minelute column (Qiagen) and electrophoresed in 0.7% agarose in the absence of ethidium bromide. DNA was blotted onto an H+ membrane and probed with random prime α-dCTP^32^ labeled p294 (Rediprime II, GE Healthcare, Chalfont St. Giles, UK).

### Immunoblotting

Cells were lysed for 1 h in IPLB (50 mM Tris-HCl, 150 mM NaCl, 2 mM EDTA, 2 mM EGTA, 25 mM NaF, 25 mM β-glycerolphosphate, 0.1 mM NaOrthovanadate, 0.2% Triton X-100, 0.3% NP-40, plus protease inhibitor cocktail (Roche, Basel, Switzerland) at 4°C and centrifuged at 13,000 rpm for 10 min. The insoluble pellet was resuspended in IPLB containing 300 mM NaCl and incubated for 30 min at 4°C. 10 U/ml of Benzonase nuclease was added, followed by incubation at RT for 30 min, and sonication for 15 min in a sonicating waterbath. ORC1 antibodies raised against the N or C terminus (N17 and H80 respectively), Orc4 (L-15), Orc6 (FL-252), Cdc6 (180.2), Cdt1 (H-300) and HP1 (FL191) were from Santa Cruz Cruz (Santa Cruz, California). Histone H3 (tri-methyl K9, ab8898) was from Abcam (Cambridge UK).

### Chondroinduction

Patient-derived hTERT immortalized fibroblasts were chondroinduced by seeding in micromass culture (2×10^5^ cells/well) onto 24 well plates coated with the chondrogenic proteoglycan aggrecan (Sigma-Aldrich). Plates were prepared using 20 µg of aggrecan/well, dried overnight at around 37°C. Aggregate sizes were measured using light microscope images (40× magnification) using Adobe Photoshop (arbitrary units, lower cut-off point at the single cell size approximately).

Semi-quantitative RT-PCR (26 cycles) for VEGFA was performed using the ProtoScript AMV LongAmp Taq RT-PCR Kit (New England Biolabs) using the following primer sets:

*VEGFA:*

Forward: 5′-GTCTTGGGTGCATTGGAGCC-3′

Reverse: 5′-CCTCGGCTTGTCACATCTGC-3′

*ELP4:*

Forward: 5′-AAGAGGATCCTGCCAACATTT-3′

Reverse: 5′-AGGATTGGATCCATCAAATCC-3′

qRT-PCR for *COL1A1* analysis was carried out using the QuantiFast SYBR Green PCR Kit and the following QuantiTect Primers (Qiagen):

*COL1A1* (NM_000088): Hs_COL1A1_1_SG (cat no. QT0037793).

*GAPDH* (NM_002046): Hs_GAPDH_1_SG (cat no. QT00079247)

Reactions containing 12.5 µl SYBR Green PCR Master Mix, 2.5 µl 10× Primer assay mix, 5 µl RNAse-free water and 5 µl template cDNA to a final volume of 25 µl were prepared in duplicate. Cycling was carried out using the Stratagene Mx3005P QPCR System. Cycling conditions: reactions were heated to 95°C for 5 minutes, followed by 40 cycles of 95°C for 10 seconds and 60°C for 30 seconds. Reactions were then heated up to 95°C for a further 1 minute and incubated at 55°C for 30 seconds. For siRNA-mediated knockdown, Smartpool (Dharmacon, Lafayette, Colorado) oligonucleotides were transfected using Metafectene-Pro Transfection Reagent (Biontex, Munich, Germany) and 48 hrs later cells were seeded onto aggrecan coated plates in duplicate for chondroinduction as described above.

## Supporting Information

Figure S1 Sensenbrenner syndrome and PCNT deficient fibroblasts have an increased centrosome copy number. a) Exponentially growing primary fibroblasts with the indicated deficiency (ORC1, IFT43, WDR35 or PCNT) were stained with anti-γ-tubulin to allow visualisation of centrosomes. Cells with >2 centrosomes were scored as defective. In a previous study, we showed that PCNT deficient cells have increased supernumerary centrosomes [36]. In this previous study, nocodozole was added to prevent cell cycle progression and it was possible, that this treatment enhanced centrosome abnormalities. In this study, exponentially growing cells were scored without nocodozole treatment. b–c) Exponentially growing cells were stained with anti-γ-tubulin and anti-CPAP to allow visualisation of centrosomes and centrioles, respectively. Cells with >2 centrosomes or >4 centrioles were scored as defective. An example of multiple centrosomes/centrioles in ORC1 deficient cells is shown in c).
(TIF)

Figure S2 Complementation of the defect in ciliogenesis and Smo localisation in ORC1-deficient patients cells expressing Gfp^+^-ORC1 cDNA. ORC1hTERT fibroblasts were transfected with GFP-tagged ORC1 cDNA and either cilia formation (panel a) or Smo localisation to cilia after SAG addition (panel b and c) examined in cells assessed to be GFP^+^. To detect GFP positive cells anti-GFP antibodies were utilised. The asterisk denotes GFP^+^ cells. In panel A, a GFP^+^ cell is shown together with rescued cilia formation. In panel B, two GFP^−^ cells are shown with no cilia formation or smo localisation. In panel C, two GFP^+^ cells are shown. Smo localisation at the cilia is evident in both cells with a zoomed overlay shown in the right panel. Although Smo and GFP both stain in the red channel, the Smo localisation can be distinguished above the GFP background staining.
(TIF)

Figure S3 Cell cycle exit after serum withdrawal. Control and ORC1 deficient fibroblasts or control cells treated with the indicated siRNA were depleted of serum for the times indicated then processed for immunofluorescence. G2 phase cells were detected with antibodies raised against CenPF, mitotic cells with phospho-Histone H3, active G1 with phospho-Rb and S phase with BrdU. Both cell populations exited the cell cycle with similar kinetics.
(TIF)

Figure S4 Cells were induced to enter G0 phase following serum depletion for 7 days. Serum was then re-added and the fraction of BrdU^+^ S phase cells monitored at the indicated times. The delay in S phase entry seen in ORC1 deficient cells is diminished after starvation for 7 days.
(TIF)

Table S1 The mutations in genes encoding origin licensing components in the MGS patients. The table describes the mutations in the MGS patients and some of their clinical features.
(PDF)
