# Supplementary figures and images for: Deficiency in Origin Licensing Proteins Impairs Cilia Formation: Implications for the Aetiology of Meier-Gorlin Syndrome
Source: PLoS Genet. 2013 Mar 14;9(3):e1003360. doi: 10.1371/journal.pgen.1003360 (PMC3597520; doi:10.1371/journal.pgen.1003360)

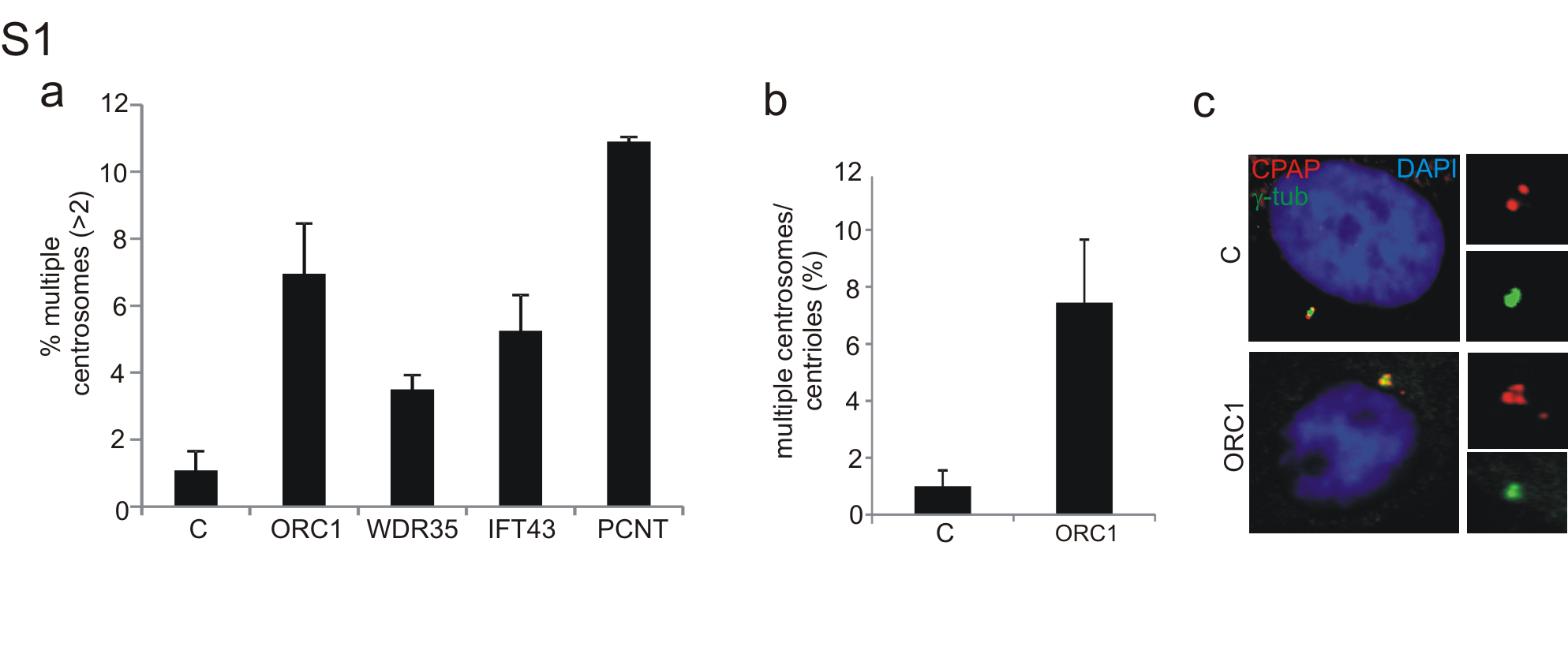

Supplement: Figure S1 — Sensenbrenner syndrome and PCNT deficient fibroblasts have an increased centrosome copy number. a) Exponentially growing primary fibroblasts with the indicated deficiency (ORC1, IFT43, WDR35 or PCNT) were stained with anti-γ-tubulin to allow visualisation of centrosomes. Cells with >2 centrosomes were scored as defective. In a previous study, we showed that PCNT deficient cells have increased supernumerary centrosomes [36]. In this previous study, nocodozole was added to prevent cell cycle progression and it was possible, that this treatment enhanced centrosome abnormalities. In this study, exponentially growing cells were scored without nocodozole treatment. b–c) Exponentially growing cells were stained with anti-γ-tubulin and anti-CPAP to allow visualisation of centrosomes and centrioles, respectively. Cells with >2 centrosomes or >4 centrioles were scored as defective. An example of multiple centrosomes/centrioles in ORC1 deficient cells is shown in c). (TIF) [file pgen.1003360.s001.tif]

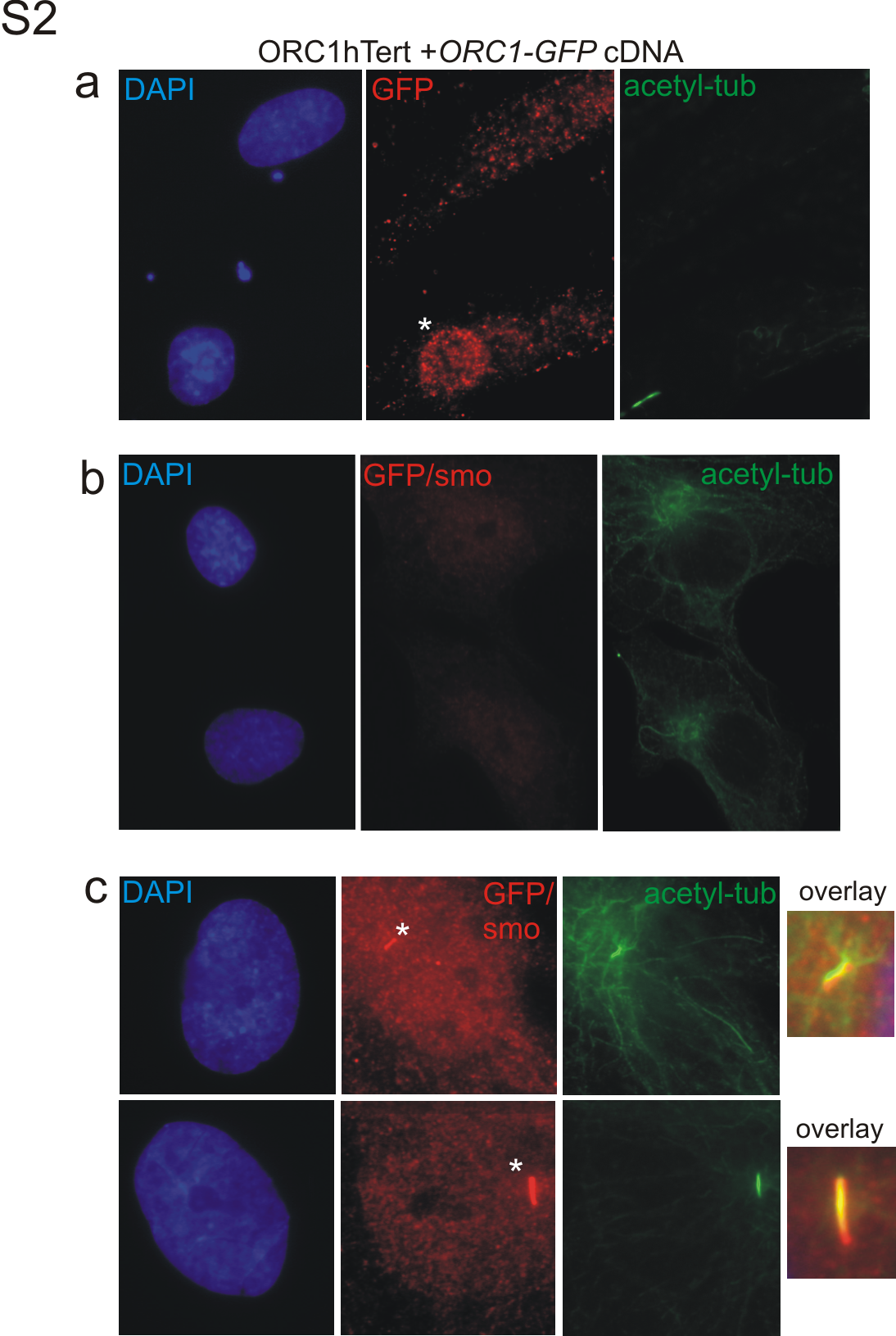

Supplement: Figure S2 — Complementation of the defect in ciliogenesis and Smo localisation in ORC1-deficient patients cells expressing Gfp+-ORC1 cDNA. ORC1hTERT fibroblasts were transfected with GFP-tagged ORC1 cDNA and either cilia formation (panel a) or Smo localisation to cilia after SAG addition (panel b and c) examined in cells assessed to be GFP+. To detect GFP positive cells anti-GFP antibodies were utilised. The asterisk denotes GFP+ cells. In panel A, a GFP+ cell is shown together with rescued cilia formation. In panel B, two GFP− cells are shown with no cilia formation or smo localisation. In panel C, two GFP+ cells are shown. Smo localisation at the cilia is evident in both cells with a zoomed overlay shown in the right panel. Although Smo and GFP both stain in the red channel, the Smo localisation can be distinguished above the GFP background staining. (TIF) [file pgen.1003360.s002.tif]

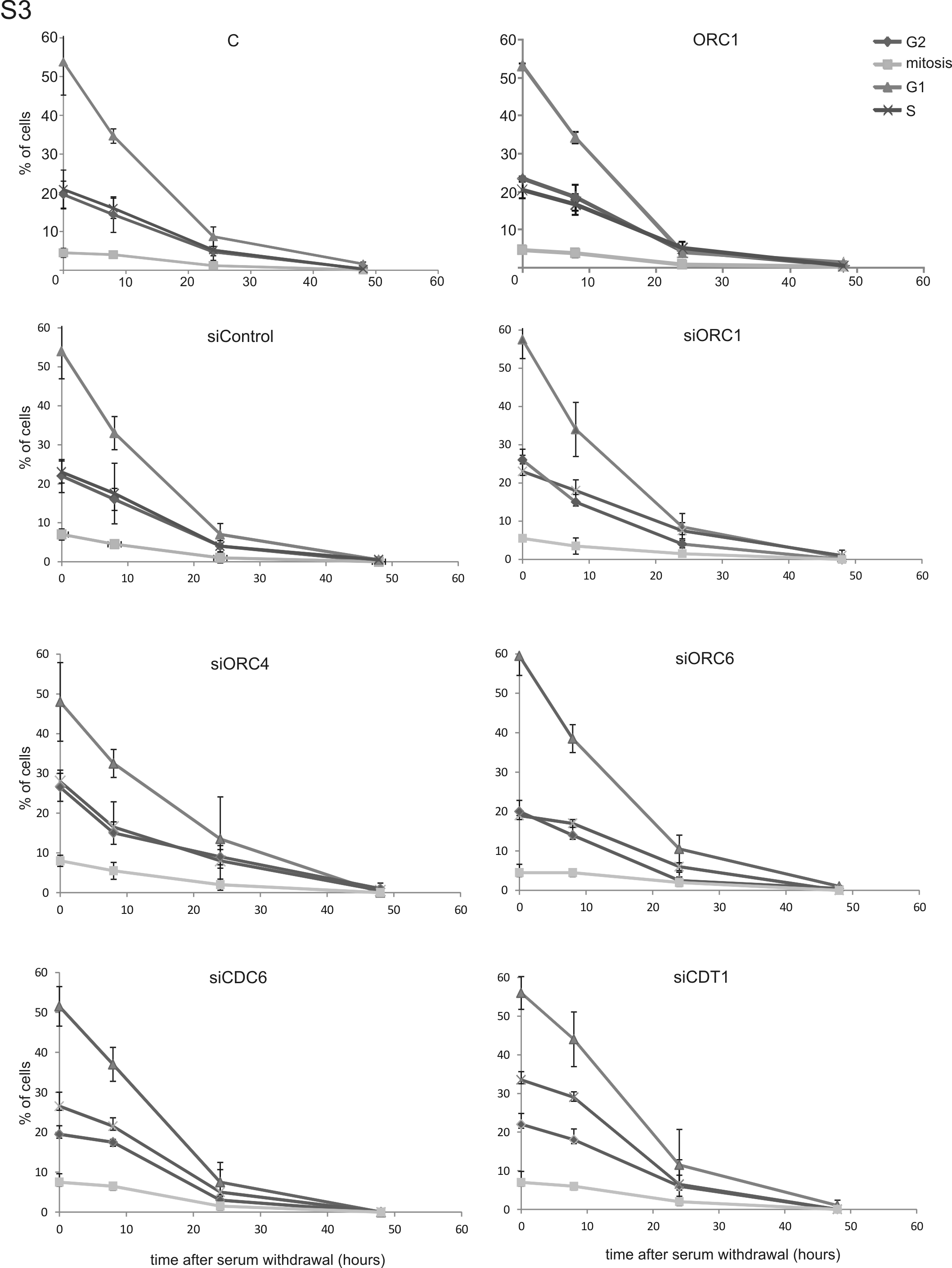

Supplement: Figure S3 — Cell cycle exit after serum withdrawal. Control and ORC1 deficient fibroblasts or control cells treated with the indicated siRNA were depleted of serum for the times indicated then processed for immunofluorescence. G2 phase cells were detected with antibodies raised against CenPF, mitotic cells with phospho-Histone H3, active G1 with phospho-Rb and S phase with BrdU. Both cell populations exited the cell cycle with similar kinetics. (TIF) [file pgen.1003360.s003.tif]

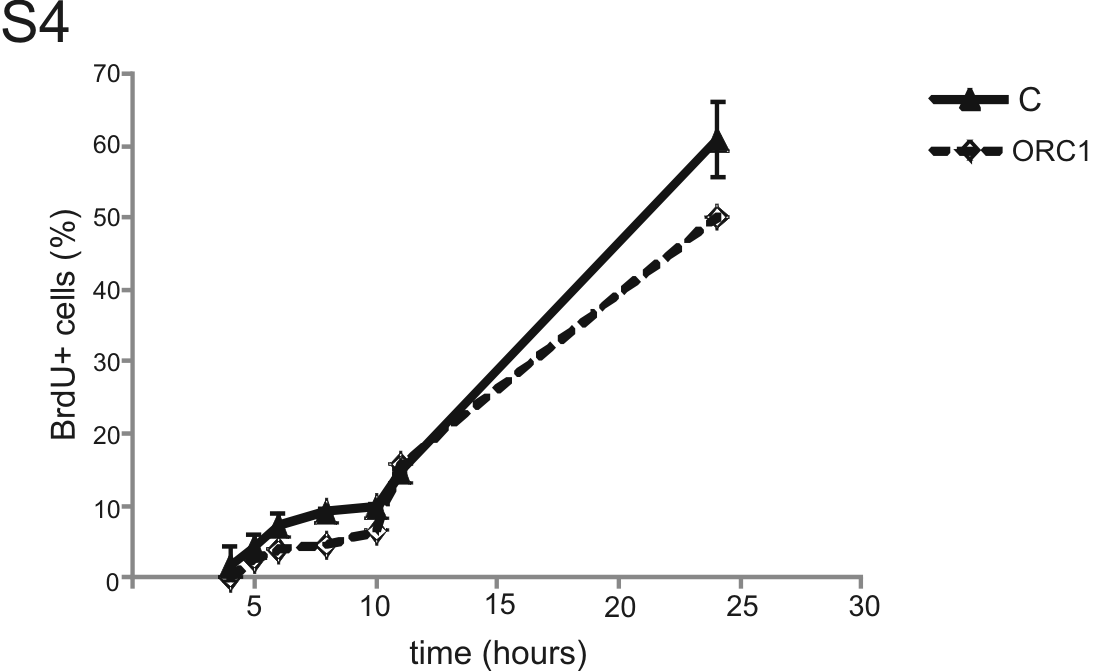

Supplement: Figure S4 — Cells were induced to enter G0 phase following serum depletion for 7 days. Serum was then re-added and the fraction of BrdU+ S phase cells monitored at the indicated times. The delay in S phase entry seen in ORC1 deficient cells is diminished after starvation for 7 days. (TIF) [file pgen.1003360.s004.tif]
