# Supplementary material for: Deficiency in Origin Licensing Proteins Impairs Cilia Formation: Implications for the Aetiology of Meier-Gorlin Syndrome
Source: PLoS Genet. 2013 Mar 14;9(3):e1003360. doi: 10.1371/journal.pgen.1003360 (PMC3597520; doi:10.1371/journal.pgen.1003360)
Supplement: Table S1 — The mutations in genes encoding origin licensing components in the MGS patients. The table describes the mutations in the MGS patients and some of their clinical features. (PDF) [file pgen.1003360.s005.pdf]

Supplementary Table 1

Gene	Nucleotide Alterations	Amino Acid Alterations	Exons	Segreg-ation	Current Height (SD)	Current OFC (SD)	Microtia	Absent/small Patellae
ORC1	[c.314G>A] + [c.1999_2000delGTinsA]	R105Q + V667fsX24*	4; 13	Het	-9.6	-9.8	+	+
ORC4	c.521A>G	Y174C	8	Hom	-4.2	-2.1	+	-
ORC6	[c.257_258delTT] + [c.695A>C]	F86X + Y232S	3; 7	Het	-3.3	-1.6	+	+
CDT1	[c.1385G>A] + [c.1560C>A]	R462Q + Y520X	9; 10	Het	-1.6	+1.7	+	+
CDC6	c.968C>G	T323R	7	Hom	-4.1	-3.3	+	+

***** The mutational change in one allele lies within the ORC1 BAH domain and is predicted to be functionally impacting. The mutation in the second allele is a frameshift mutation in the C-terminal region. The cells have normal ORC1 protein expression but diminished chromatin bound ORC1.
